# Computational identification of ubiquitylation sites from protein sequences

**DOI:** 10.1186/1471-2105-9-310

**Published:** 2008-07-15

**Authors:** Chun-Wei Tung, Shinn-Ying Ho

**Affiliations:** 1 Institute of Bioinformatics, National Chiao Tung University, Hsinchu 300, Taiwan; 2 Department of Biological Science and Technology, National Chiao Tung University, Hsinchu 300, Taiwan

## Abstract

**Background:**

Ubiquitylation plays an important role in regulating protein functions. Recently, experimental methods were developed toward effective identification of ubiquitylation sites. To efficiently explore more undiscovered ubiquitylation sites, this study aims to develop an accurate sequence-based prediction method to identify promising ubiquitylation sites.

**Results:**

We established an ubiquitylation dataset consisting of 157 ubiquitylation sites and 3676 putative non-ubiquitylation sites extracted from 105 proteins in the UbiProt database. This study first evaluates promising sequence-based features and classifiers for the prediction of ubiquitylation sites by assessing three kinds of features (amino acid identity, evolutionary information, and physicochemical property) and three classifiers (support vector machine, *k*-nearest neighbor, and NaïveBayes). Results show that the set of used 531 physicochemical properties and support vector machine (SVM) are the best kind of features and classifier respectively that their combination has a prediction accuracy of 72.19% using leave-one-out cross-validation.

Consequently, an informative physicochemical property mining algorithm (IPMA) is proposed to select an informative subset of 531 physicochemical properties. A prediction system UbiPred was implemented by using an SVM with the feature set of 31 informative physicochemical properties selected by IPMA, which can improve the accuracy from 72.19% to 84.44%. To further analyze the informative physicochemical properties, a decision tree method C5.0 was used to acquire if-then rule-based knowledge of predicting ubiquitylation sites. UbiPred can screen promising ubiquitylation sites from putative non-ubiquitylation sites using prediction scores. By applying UbiPred, 23 promising ubiquitylation sites were identified from an independent dataset of 3424 putative non-ubiquitylation sites, which were also validated by using the obtained prediction rules.

**Conclusion:**

We have proposed an algorithm IPMA for mining informative physicochemical properties from protein sequences to build an SVM-based prediction system UbiPred. UbiPred can predict ubiquitylation sites accompanied with a prediction score each to help biologists in identifying promising sites for experimental verification. UbiPred has been implemented as a web server and is available at .

## Background

Ubiquitylation (also called ubiquitination) is an important mechanism of post-translational modification that ubiquitin will be linked to specific lysine residues of target proteins by forming isopeptide bonds. Three enzymes including activating enzyme (E1), conjugating enzyme (E2), and ubiquitin ligase (E3) are involved in the ubiquitylation process. Another enzyme E4 can help to stabilize and extend polyubiquitin chain [1,2]. The first discovered function of ubiquitylation is to target proteins for subsequent degradation by the ATP-dependent ubiquitin-proteasome system. Subsequently, many regulatory functions of ubiquitylation were discovered including the regulation of DNA repair and transcription, control of signal transduction, and implication of endocytosis and sorting [1,2].

Because of the important regulatory roles of ubiquitylation, numerous methods were developed to purify ubiquitylated proteins [3]. Also, the growing number of studies of large-scale identification of ubiquitylated proteins and analysis of ubiquitin-related proteome reflect the importance of identifying ubiquitylation proteins and sites [4-9]. The three steps affinity purification, proteolytic digestion, and analysis using mass spectrometry were applied in most of these studies [10]. These works cost a lot of experimental efforts. Therefore, this study focuses on the computational identification of ubiquitylation sites from protein sequences by developing an accurate prediction method.

Using both informative features and an appropriate classifier is essential to design an effective system for prediction of ubiquitylation sites. In the past, numerous sequence-derived features have been proposed to discriminate protein or residue functions. For example, the AutoMotif server utilized six kinds of features and support vector machine (SVM) to predict post translational modifications [11]. The POPI server used physicochemical properties as efficient features to predict peptide immunogenicity [12]. In this study, three kinds of useful features which can be extracted from protein sequences are evaluated: conventional amino acid identity [11,13], evolutionary information [14,15], and physicochemical property [12,16]. At the same time, three machine learning classifiers, *k*-nearest neighbor, NaïveBayes, and SVM are also evaluated.

We established an ubiquitylation dataset (UBIDATA) consisting of 157 ubiquitylation sites and 3676 putative non-ubiquitylation sites extracted from 105 proteins in UbiProt, a database of ubiquitylated proteins [17]. For predicting functions of a residue in a protein, it is well recognized that nearby residues will influence the property and structure of a central residue. The environmental information will be useful to enhance prediction accuracy that is extensively used in previous studies [13-15]. We constructed ten datasets with window sizes 11, 13,..., 29 from UBIDATA to evaluate all combinations of the evaluated features and classifiers. According to the prediction accuracies of using 10-fold cross-validation (10-CV), the physicochemical property and SVM are the best kind of features and classifier, respectively.

In order to provide insights into the underlying mechanism of ubiquitylation and advance the prediction accuracy, an informative physicochemical property mining algorithm (IPMA) is proposed to further select an informative subset of 531 physicochemical properties based on an inheritable bi-objective genetic algorithm [18]. This approach to identifying a problem-dependent set of informative physicochemical properties served as input features to SVM is shown to be effective in predicting both protein subnuclear localization [16] and immunogenicity of MHC class I binding peptides [12]. By applying IPMA to mine informative physicochemical properties and tune SVM parameters while maximizing the 10-CV accuracy, a set of 31 informative physicochemical properties was obtained. Based on the informative physicochemical properties, a decision tree method C5.0 [19] was used to acquire if-then rule-based knowledge for biologists to further understand the mechanism of ubiquitylation.

A prediction system UbiPred for predicting ubiquitylation sites was implemented by utilizing the 31 informative physicochemical properties. UbiPred performs well with a prediction accuracy of 84.44% using leave-one-out cross-validation (LOOCV), compared with the SVM-based methods using amino acid identity (65.67%), evolutionary information (66.33%) and all physicochemical properties (72.19%). Besides the prediction accuracy, the receiver operating characteristic (ROC) curve is commonly used to evaluate the discrimination ability of a classifier. The larger the area under the ROC curve, the better discrimination ability a classifier has. The area under the ROC curve of UbiPred is as high as 0.85 by using the decision value of SVM as a tuning parameter. UbiPred has been implemented as a web server and is available online [20].

Because there are still many ubiquitylation sites to be discovered [4], UbiPred can predict ubiquitylation sites accompanied with a prediction score (ranged from 0 to 1) each to help biologists in selecting the most promising sites for experimental verification. By selecting the sites with scores larger than 0.85 from an independent dataset of 3424 putative non-ubiquitylation sites, 23 promising ubiquitylation sites can be identified. The *in silico *validation by using the prediction rules obtained from C5.0 provides another confirmation in identifying the 23 promising sites as ubiquitylation sites.

## Results and discussion

### Assessments of features and classifiers

The dataset UBIDATA consists of 157 ubiquitylation sites and 3676 putative non-ubiquitylation sites extracted from 105 proteins in UbiProt [17]. Ten datasets with window sizes *w *= 11, 13,..., 29 were constructed from UBIDATA to assess three kinds of sequence-based features and three classifiers: IBk (*k*-nearest neighbor classifier), NaïveBayes, and SVM (see the section Methods). In assessing the feature of physicochemical properties, all *n *= 531 properties available were used. Five versions of the classifier IBk with *k *= 1, 3,..., 9 were evaluated to find the best value of *k *for classification. For NaïveBayes, both the normal distribution and the estimated distribution were applied to evaluate prediction performances.

**Figure 1:**
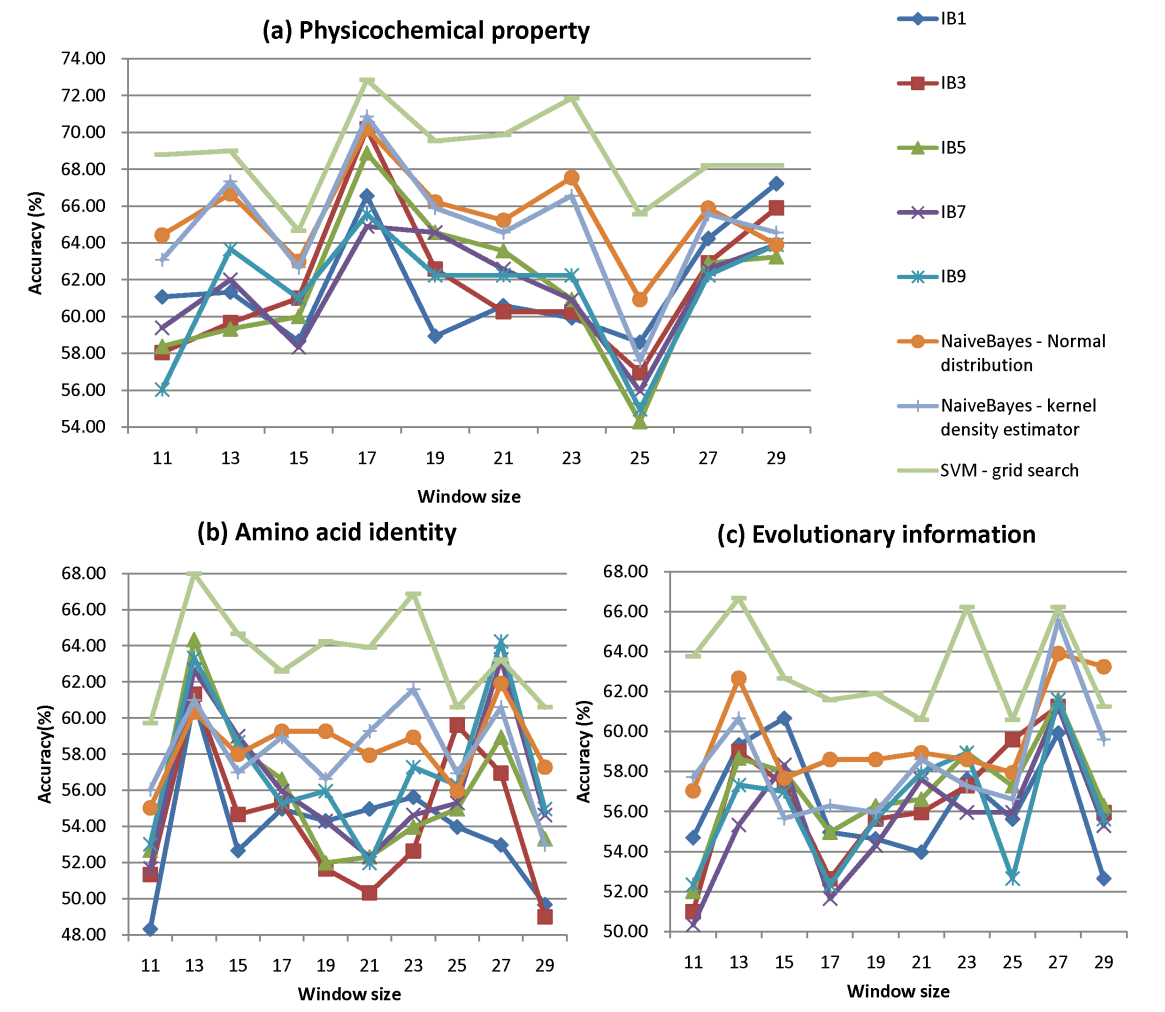
Performance comparisons among various classifiers with the three kinds of features. (a) physicochemical property, (b) amino acid identity, and (c) evolutionary information.

Figure 1 shows the accuracies of 10-CV using IBk, NaïveBayes, and SVM with the three kinds of features. For each kind of features, the SVM performs best compared with the other classifiers. The best performances of SVM using the features, amino acid identity (*w *= 13), evolutionary information (*w *= 13), and physicochemical property (*w *= 17), are 68.00%, 66.67%, and 72.85%, where the corresponding values of SVM parameters (*C*, γ) are (2, 2^-2^), (1, 2^-7^) and (1, 2^-4^), respectively. The results reveal that the physicochemical property is the best kind of features to the SVM for predicting ubiquitylation sites, compared with amino acid identity and evolutionary information.

**Figure 2:**
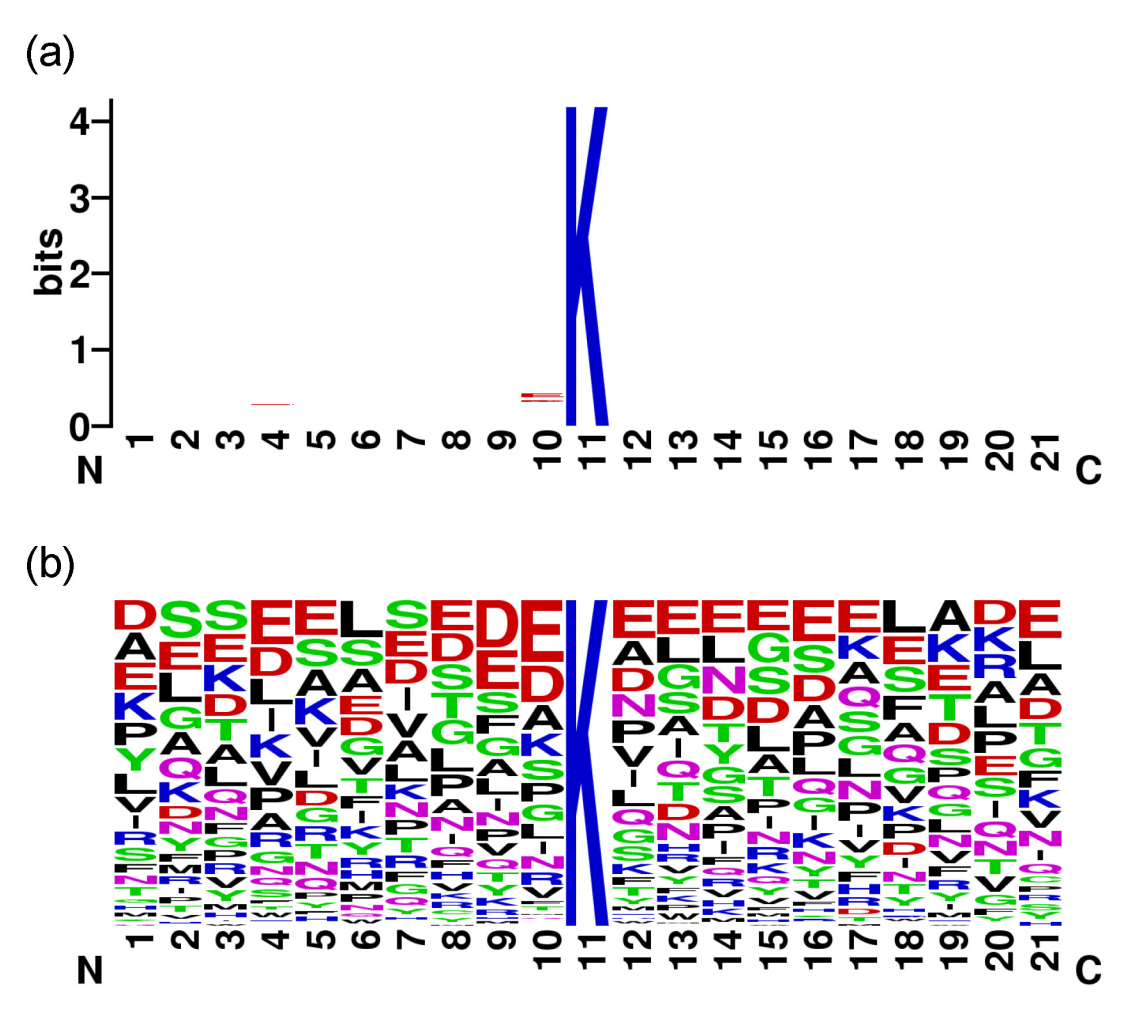
The sequence logo of the 151 positive samples with *w *= 21. (a) information content and (b) frequency plot.

Figure 2 shows the sequence logo of the 151 positive samples with *w *= 21 generated by the WebLogo tool [21]. The sequence logo with low information content reveals disadvantages of the SVM using the two position-based features, amino acid identity and evolutionary information, compared with the non-position based features, physicochemical properties using averaged measurement of amino acids in a sequence.

### Informative physicochemical properties

Most of the 531 physicochemical properties may be irrelevant features or even interfere with prediction of the SVM classifier. Therefore, it is important to mine informative physicochemical properties for advancing the prediction accuracy. IPMA determines a feature set of *r *informative physicochemical properties and the values of SVM parameters (*C *and γ) for a given window size *w*. Because of the non-deterministic nature of IPMA, the obtained solutions would be different for each run. To obtain the features with robust performance, 30 independent runs of IPMA were performed for each window size *w*.

**Figure 3:**
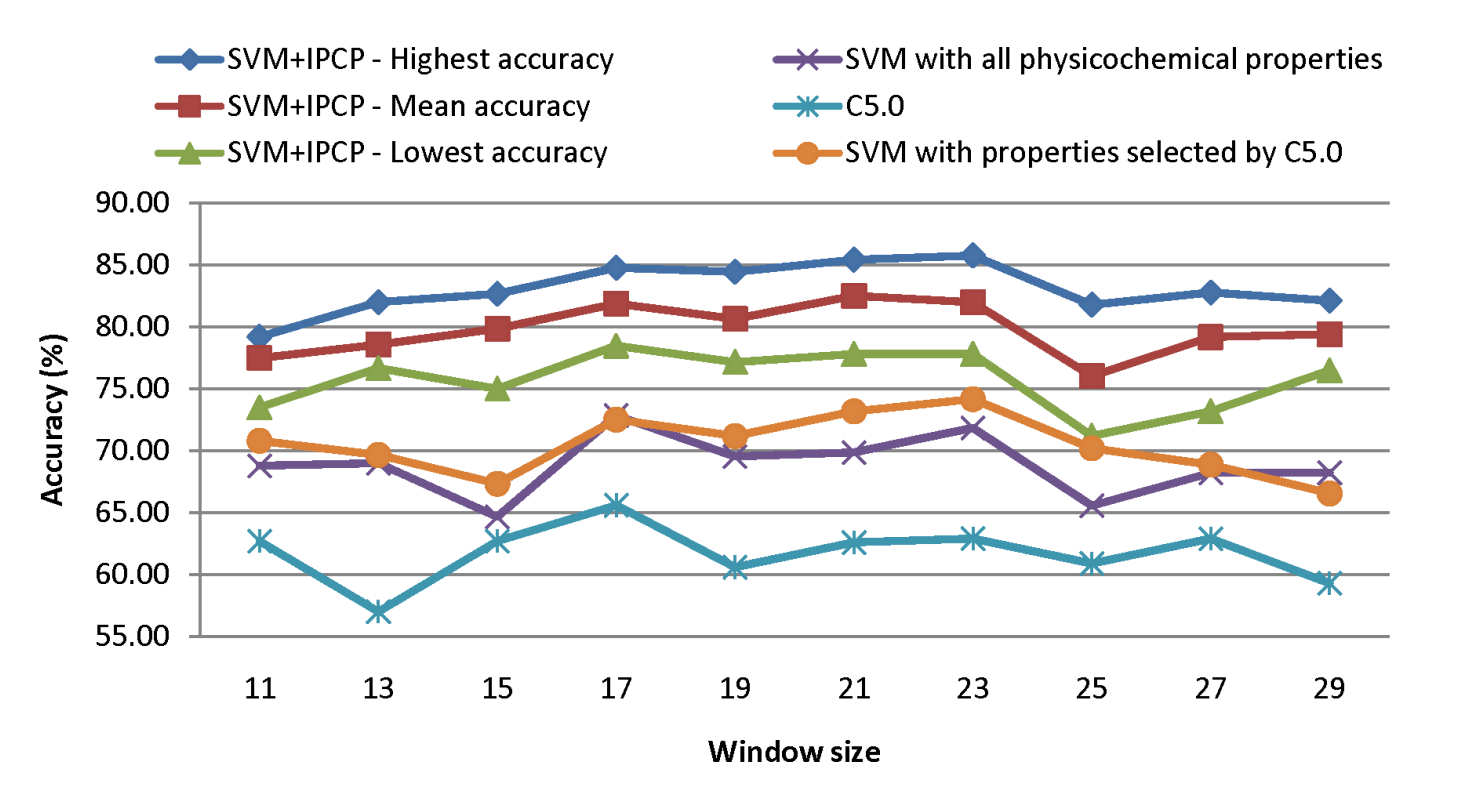
Performance comparisons between the SVM with informative physicochemical properties (SVM+IPCP) and other compared classifiers.

The highest, mean, and lowest prediction accuracies of IPMA using 10-CV are shown in Fig. 3. For comparison, the decision tree method C5.0 [19] with the ability of feature selection based on information gain was also evaluated. The accuracies of C5.0 and SVM with the properties selected by C5.0 for various window sizes are also given in Fig. 3. For all window sizes, the accuracies of SVM using informative physicochemical properties mined by IPMA are better than those of C5.0, SVM using all 531 physicochemical properties, and SVM using the C5.0-selected properties. Considering the mean accuracies of SVM with informative physicochemical properties in Fig. 3, the best window size is *w *= 21.

**Figure 4:**
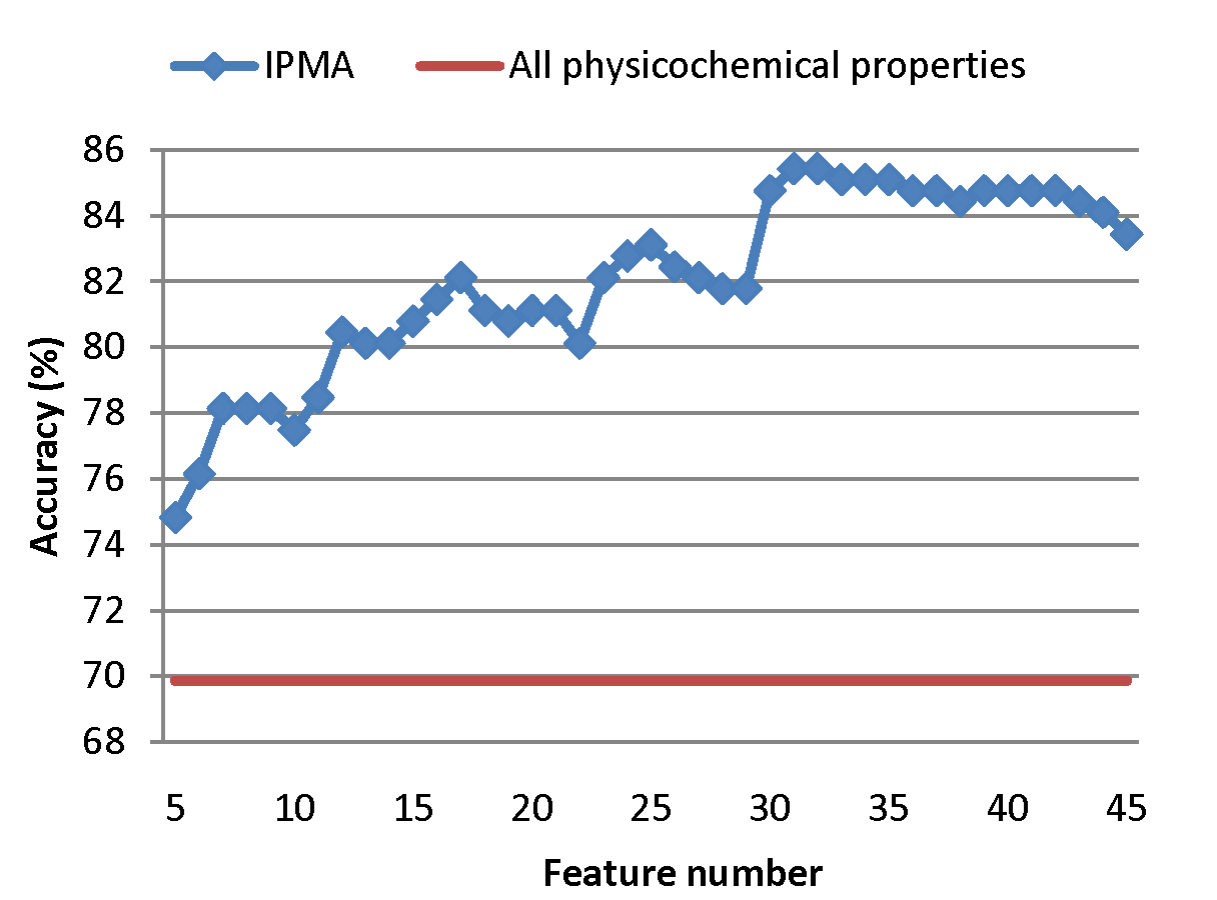
The best 10-CV accuracies of prediction using SVM with the window size 21 for various numbers of features (properties) selected by IPMA from 30 independent runs.

Figure 4 shows the best 10-CV accuracies of using IPMA with *w *= 21 for various numbers of features from 30 independent runs. The accuracy of *w *= 21 can be improved from 69.87% to 85.43% by using *m *= 31 out of *n *= 531 physicochemical properties, where the values of SVM parameters are *C *= 4 and γ = 0.5. The 31 informative physicochemical properties constitute a good feature set obtained by considering the inter-correlation among properties.

The quantified effectiveness of individual physicochemical properties on prediction is useful to characterize the ubiquitylation mechanism by physicochemical properties. Orthogonal experimental design with factor analysis [22,23] can be used to estimate the individual effects of physicochemical properties according to the value of main effect difference (MED) [12,16]. The property with the largest value of MED is the most effective in predicting ubiquitylation sites.

According to MED, the 31 informative properties are ranked and their descriptions are shown in Table 1. The most effective property with *MED *= 31.79 is NADH010102 denoting "hydropathy scale based on self-information values in the two-state model of 9% accessibility". The least effective properties with *MED *= 1.32 are NAKH900101 and QIAN880129 denoting "amino acid composition of total protein" and "weights for coil at the window position of -4", respectively. The ranked informative physicochemical properties provide valuable information to biologists for further experimental verification.

**Table 1:** The 31 informative physicochemical properties mined by IPMA.

AAindex identity	Description	MED
NADH010102	Hydropathy scale based on self-information values in the two-state model of 9% accessibility	31.79
BROC820102	Retention coefficient in HFBA	29.80
MEIH800102	Average reduced distance for side chain	28.48
LEVM780101	Normalized frequency of alpha-helix, with weights	25.17
GUYH850104	Apparent partition energies calculated from Janin index	23.84
CORJ870101	NNEIG index	23.18
RACS770102	Average reduced distance for side chain	22.52
GEOR030108	Linker propensity from helical (annotated by DSSP) dataset	22.52
HARY940101	Mean volumes of residues buried in protein interiors	21.85
GRAR740102	Polarity	19.87
GUYH850105	Apparent partition energies calculated from Chothia index	19.87
MEIH800103	Average side chain orientation angle	17.88
KRIW790102	Fraction of site occupied by water	17.88
LEVM780106	Normalized frequency of reverse turn, unweighted	14.57
BULH740102	Apparent partial specific volume	13.25
FAUJ880101	Graph shape index	11.92
PUNT030102	Knowledge-based membrane-propensity scale from 3D_Helix in MPtopo databases	10.60
HUTJ700103	Entropy of formation	9.93
EISD840101	Consensus normalized hydrophobicity scale	8.61
CEDJ970105	Composition of amino acids in nuclear proteins (percent)	7.28
ZIMJ680102	Bulkiness	7.28
CEDJ970103	Composition of amino acids in membrane proteins (percent)	5.96
CHOC760103	Proportion of residues 95% buried	5.30
CEDJ970102	Composition of amino acids in anchored proteins (percent)	5.30
ROSM880102	Side chain hydropathy, corrected for solvation	4.64
BROC820101	Retention coefficient in TFA	4.64
FAUJ830101	Hydrophobic parameter pi	1.99
NAKH920101	AA composition of CYT of single-spanning proteins	1.99
ZHOH040102	The relative stability scale extracted from mutation experiments	1.99
NAKH900101	AA composition of total proteins	1.32
QIAN880129	Weights for coil at the window position of -4	1.32

### Knowledge of data mining

**Figure 5:**
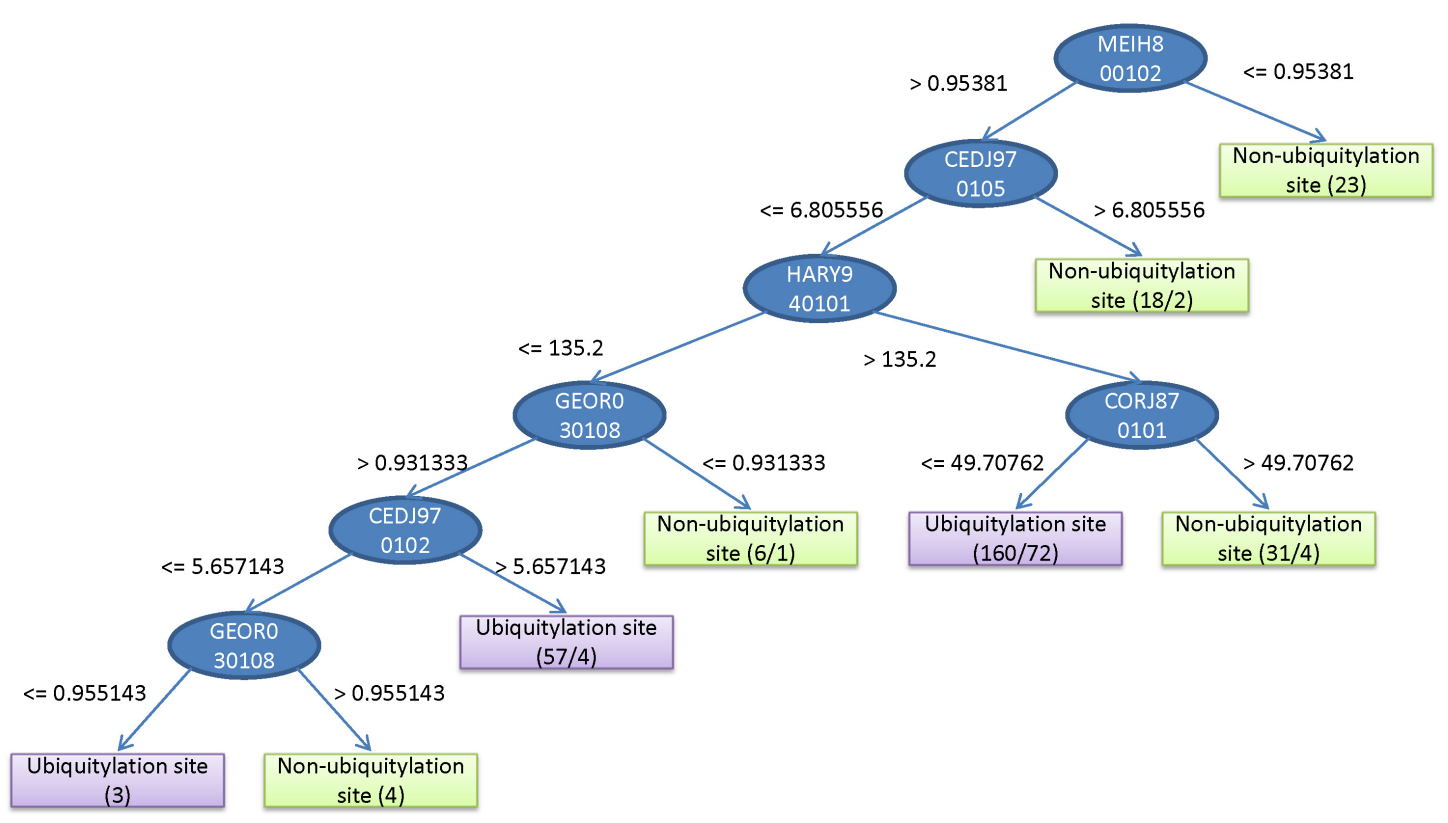
The derived decision tree by using C5.0 and the features of informative physicochemical properties for classification of ubiquitylation sites.

Although the prediction accuracy of SVM is rather high compared with the other classifiers evaluated, it is not easy for biologist to interpret the prediction rules. In order to acquire interpretable knowledge from experimental data, C5.0 was applied to construct a compact decision tree by using the 31 informative physicochemical properties selected by IPMA on the whole training dataset. Figure 5 shows a constructed decision tree by C5.0. By utilizing this decision tree to classify the whole training dataset, the accuracy is 72.5%. This decision tree can be directly converted into a set of eight interpretable rules [19], consisting of three and five if-then rules for ubiquitylation sites and non-ubiquitylation sites, respectively.

To obtain rather simple rules for easy interpretation, five concise if-then rules obtained from C5.0 are shown in Table 2. The first rule with the highest confidence value 0.96 can be interpreted as 'given a peptide with a central residue lysine (*w *= 21), if the average reduced distance for side chain [24] (property MEIH800102) is less than or equal to 0.95381, then the residue is a non-ubiquitylation site with a confidence value 0.96'. This rule covers 23 sites in the training dataset and no site is misclassified by this rule.

**Table 2:** Five concise if-then rules with confidence larger than 0.5 obtained by using C5.0 and 31 informative physicochemical properties.

#	Rule	Confidence	Ubiquitylation sites	Covered samples	Misclassified samples
1	MEIH800102 < = 0.95381	0.96	N	23	0
2	HARY940101 > 135.2 AND CORJ870101 > 49.70762	0.90	N	49	4
3	CEDJ970105 > 6.805556	0.85	N	18	2
4	GEOR030108 < = 0.931333	0.75	N	10	2
5	MEIH800102 > 0.95381	0.54	Y	279	128

There is only one of five classification rules for identifying ubiquitylation sites with a moderate confidence value 0.54. This rule means that if the average reduced distance for side chain is larger than 0.95381, then the residue is an ubiquitylation site with a confidence value 0.54. This rule reveals that the ubiquitylation sites are not easily discriminated from non-ubiquitylation sites. Furthermore, the property MEIH800102 plays an important role in predicting ubiquitylation sites. Examining the MED value (28.48) of MEIH800102 in Table 1, it is rather consistent that MEIH800102 is an informative property with a rank 3.

The second rule means that if the mean volume of residues buried in protein interiors [25] (property HARY940101) is larger than 135.2 and the NNEIG index [26] (property CORJ870101) is larger than 49.70762, then the residue is a non-ubiquitylation site with a confidence value 0.90'. This rule covers 49 samples in the training dataset and 4 of them are misclassified by this rule.

The third rule indicates that if the composition of amino acids in nuclear proteins (percent) [27] is larger than 6.805556, then the residue is a non-ubiquitylation site with a confidence value 0.85'. This rule covers 18 samples in the training dataset and 2 of them are misclassified.

The fourth rule indicates that if the linker propensity from helical (annotated by DSSP) dataset [28] is less than or equal to 0.931333, then the residue is a non-ubiquitylation site with a confidence value 0.75'. This rule covers 10 samples in the training dataset and 2 of them are misclassified.

### Prediction system UbiPred

**Figure 6:**
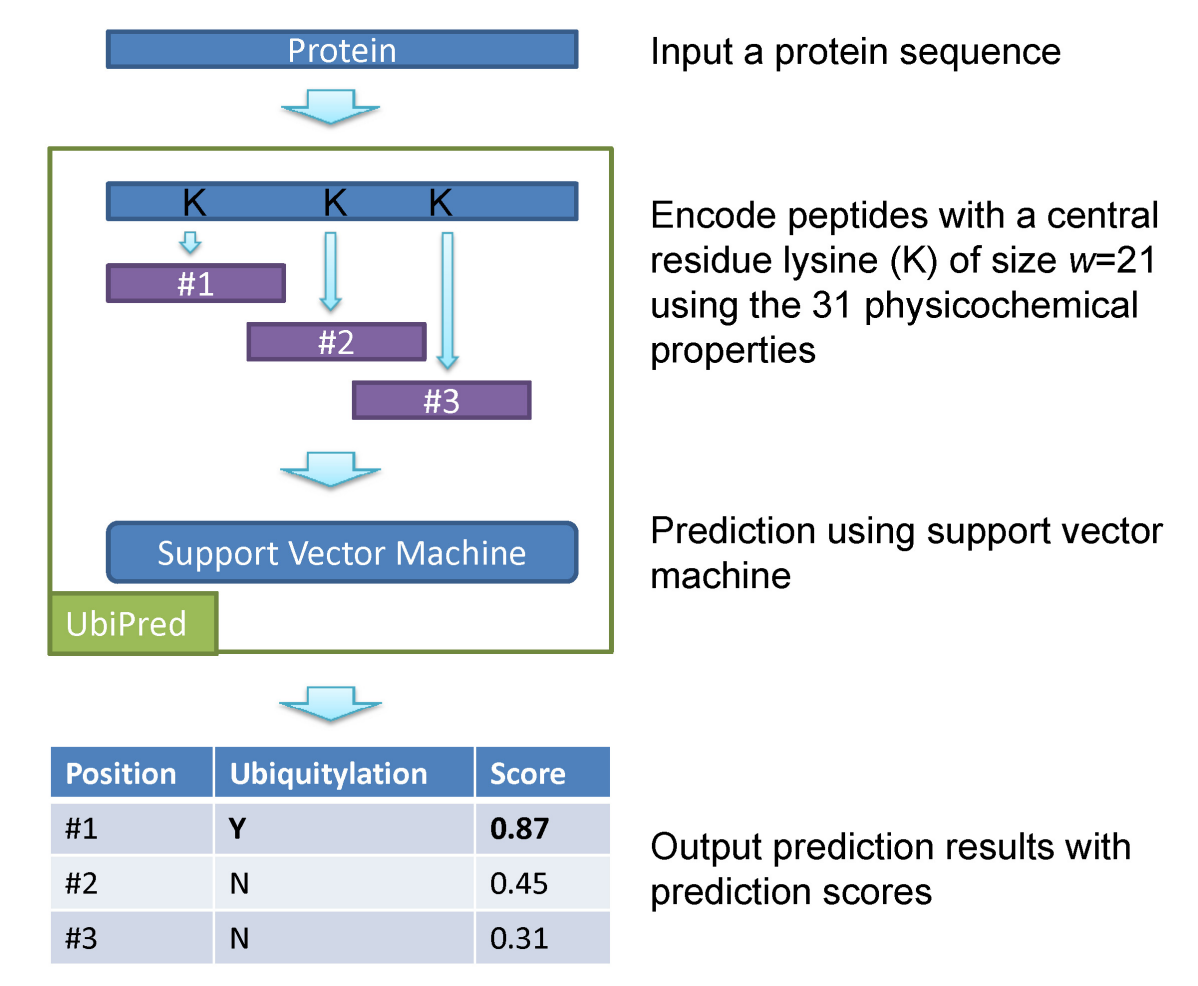
The system flow of the prediction server UbiPred.

The 31 informative physicochemical properties (shown in Table 1) with *w *= 21, *C *= 4, and γ = 0.5 were used to implement a prediction system UbiPred for identifying ubiquitylation sites. The system flow of the prediction server UbiPred is shown in Fig. 6. The input to UbiPred is a protein sequence. UbiPred will automatically encode the peptide with a central residue lysine of size *w *= 21 using the 31 informative physicochemical properties. Subsequently, the lysine residues will be annotated in terms of both ubiquitylation and a prediction score.

For comparisons with UbiPred, the same LOOCV performances of SVM using the three kinds of features: all physicochemical properties, amino acid identity, and evolutionary information are also evaluated using their corresponding best parameter settings obtained from the previous learning results, shown in Table 3.

**Table 3:** The LOOCV performances of the SVM with various kinds of features:

	Feature	Window size *w*	C	*γ*	ACC (%)	SEN (%)	SPE (%)	MCC	AUC
1	Informative physicochemical properties (UbiPred)	21	4	2^-1^	84.44	83.44	85.43	0.69	0.85
2	All physicochemical properties	17	1	2^-4^	72.19	70.86	73.51	0.44	0.74
3	Amino acid identity	13	2	2^-2^	65.67	57.33	74.00	0.32	0.70
4	Evolutionary information	13	1	2^-7^	66.33	72.00	60.67	0.33	0.71

informative physicochemical properties (UbiPred), amino acid identity, evolutionary information, and all physicochemical properties.

Four measurements were used for evaluation of prediction performances including sensitivity (SEN), specificity (SPE), accuracy (ACC), and Matthew's correlation coefficient (MCC), defined as follows: SEN = TP/(TP + FN), SPE = TN/(TN + FP), ACC = (TP + TN)/(TP + FP + TN + FN), and MCC = ((TP × TN)-(FN × FP))/((TP + FN)(TN + FP)(TP + FP)(TN + FN)), where TP, TN, FP and FN are the numbers of true positive, true negative, false positive and false negative, respectively.

**Figure 7:**
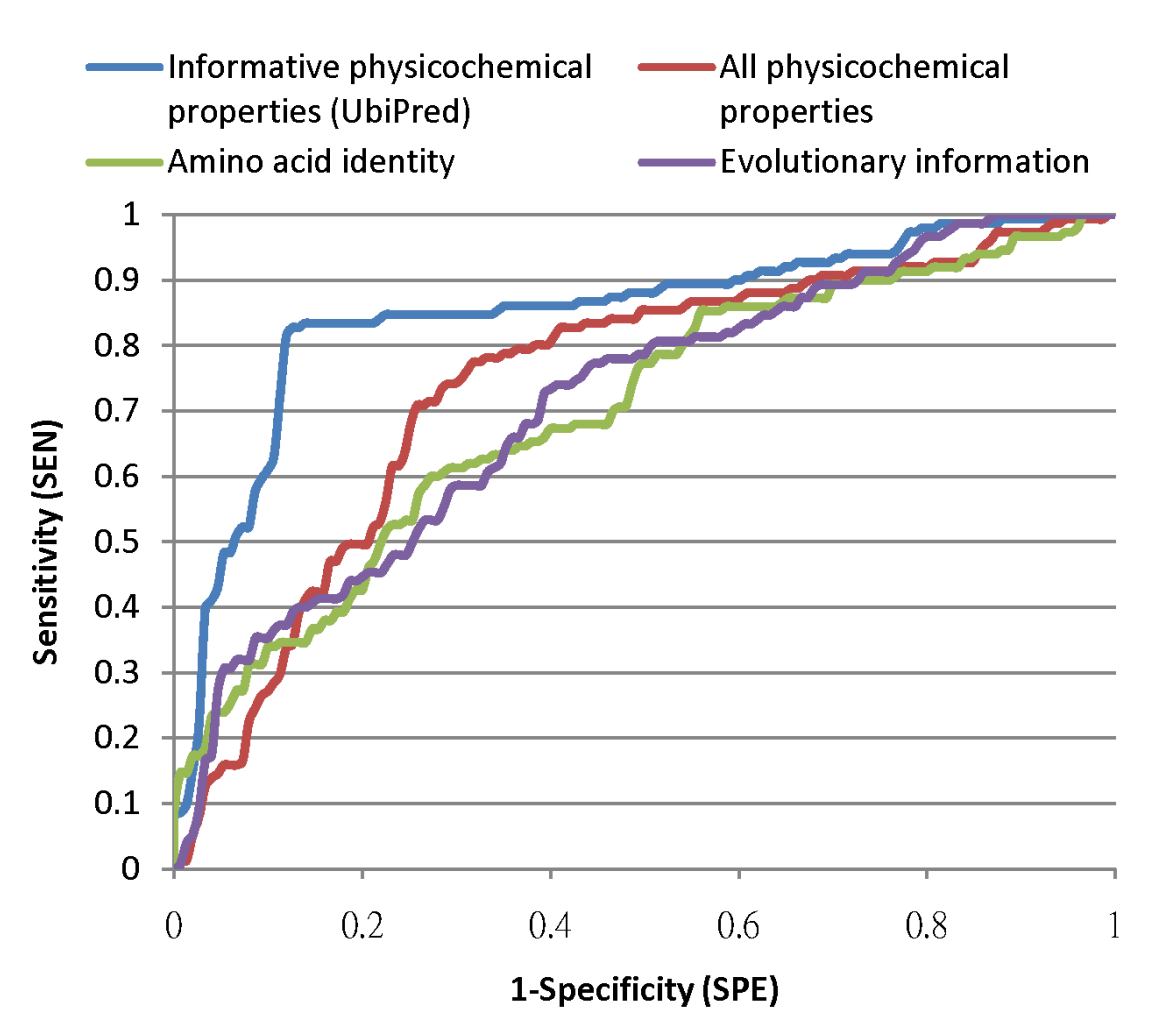
Performance comparison of SVM with various features, informative physicochemical properties (UbiPred), amino acid identity, evolutionary information, and all physicochemical properties, in terms of receiver operating characteristic curves.

UbiPred performs well with a prediction accuracy of 84.44%, compared with the SVMs with physicochemical property (72.19%), amino acid identity (65.67%) and evolutionary information (66.33%). The SEN, SPE and MCC performances of UbiPred are 83.44%, 85.43% and 0.69, respectively. To compare UbiPred with other methods in terms of robustness abilities, the nonparametric method of using a ROC curve is utilized by using the decision value of SVM as a tuning parameter. The area under the ROC curve (AUC) is calculated, as shown in Fig. 7. UbiPred with AUC = 0.85 performs well, compared with the SVM-based methods using all physicochemical properties (0.74), amino acid identity (0.70) and evolutionary information (0.71).

The problem of sequence redundancy may result in overestimation of prediction performance. To address this issue, six thresholds of sequence identity (90%, 80%,..., 40%) were applied to construct six additional datasets from the dataset of *w *= 21 by using CD-HIT [29]. The numbers of positive and negative samples of datasets with various sequence identity thresholds are shown in Table 4. By using the strictest threshold 40%, there are only 36 redundant samples and the resulting dataset consists of 145 negative samples and 121 positive samples. By applying LOOCV to evaluate prediction accuracies on these datasets, good performance (> 79%) was obtained by using SVM with the mined 31 informative physicochemical properties and SVM parameters (shown in Table 4). The results show the effectiveness of the proposed UbiPred.

**Table 4:** The LOOCV performances of the SVM with 31 informative physicochemical properties on datasets of various sequence identity thresholds.

Sequence identity threshold	Accuracy(%)	Number of positive samples	Number of negative samples
100%	84.44	151	151
90%	82.71	145	150
80%	81.72	141	149
70%	80.63	136	148
60%	81.23	131	146
50%	80.80	130	146
40%	79.70	121	145

### Screening promising ubiquitylation sites

Recently, a new experimental method was proposed with 2.4-fold increase in the number of identified ubiquitylation sites, compared with previous methods [4]. It implies that there may be still many undiscovered ubiquitylation sites. To identify promising ubiquitylation sites from putative non-ubiquitylation sites, a scoring method is designed by normalizing the range of the decision values of SVM obtained from the training dataset of *w *= 21 into the range [0, 1] of prediction scores. Normally, the default threshold value 0 used by the SVM classifier for discriminating ubiquitylation sites from non-ubiquitylation sites is mapped to a prediction score 0.5. The site with a prediction score close to 1 has a high possibility to be an ubiquitylation site. If the high prediction score 0.85 instead of 0.5 was adopted when classifying the peptides in the training dataset for all window sizes, there would be no false positive.

**Figure 8:**
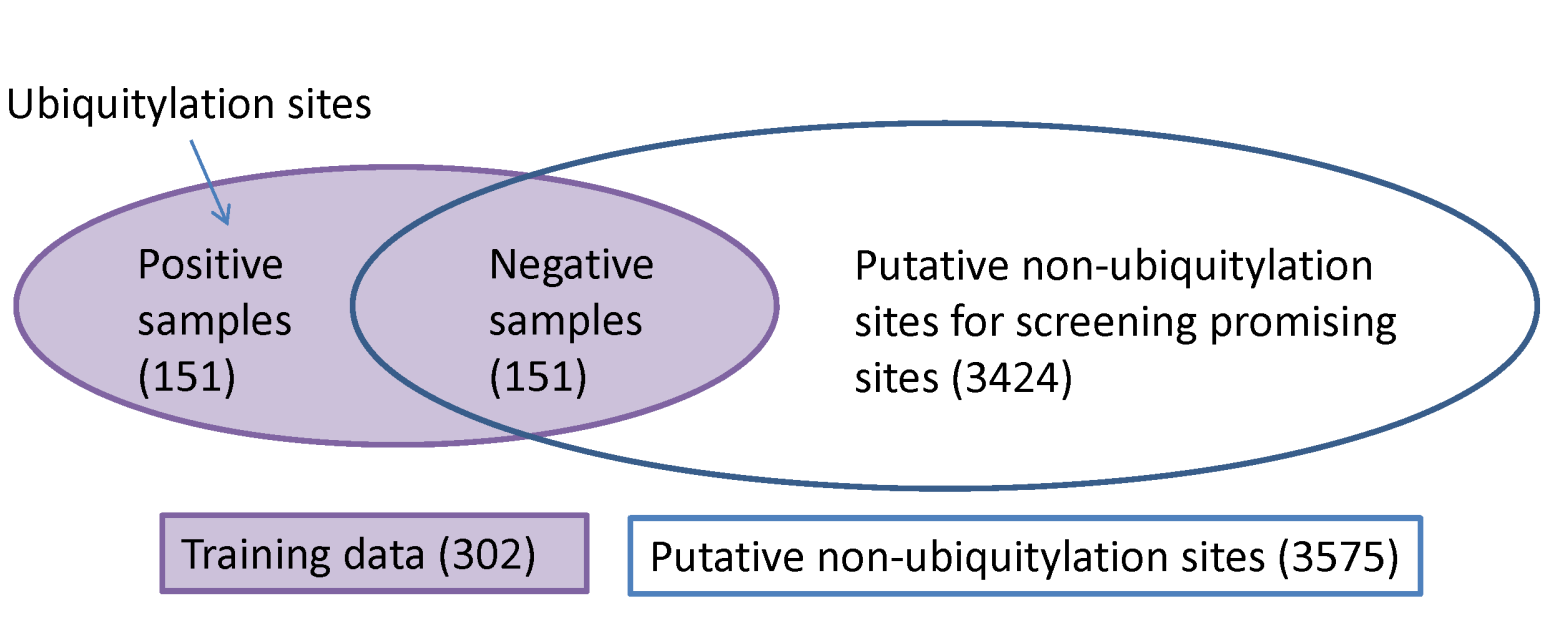
The schema for illustrating the training data (302 samples) and the independent dataset (3424 putative non-ubiquitylation sites) using *w *= 21 as an example.

**Figure 9:**
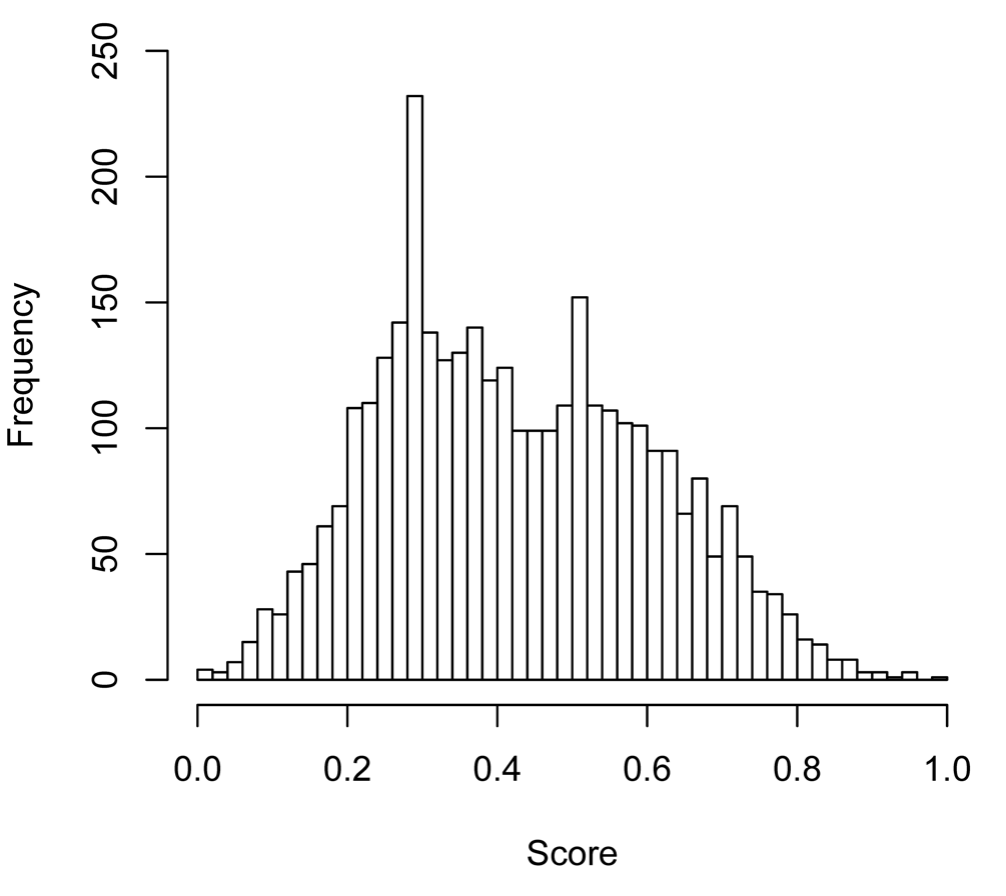
**Histogram result of UbiPred using prediction scores from evaluating 3424 putative non-ubiquitylation sites in an independent dataset**. The site with a score close to 1 has a high possibility to be an ubiquitylation site.

**Figure 10:**
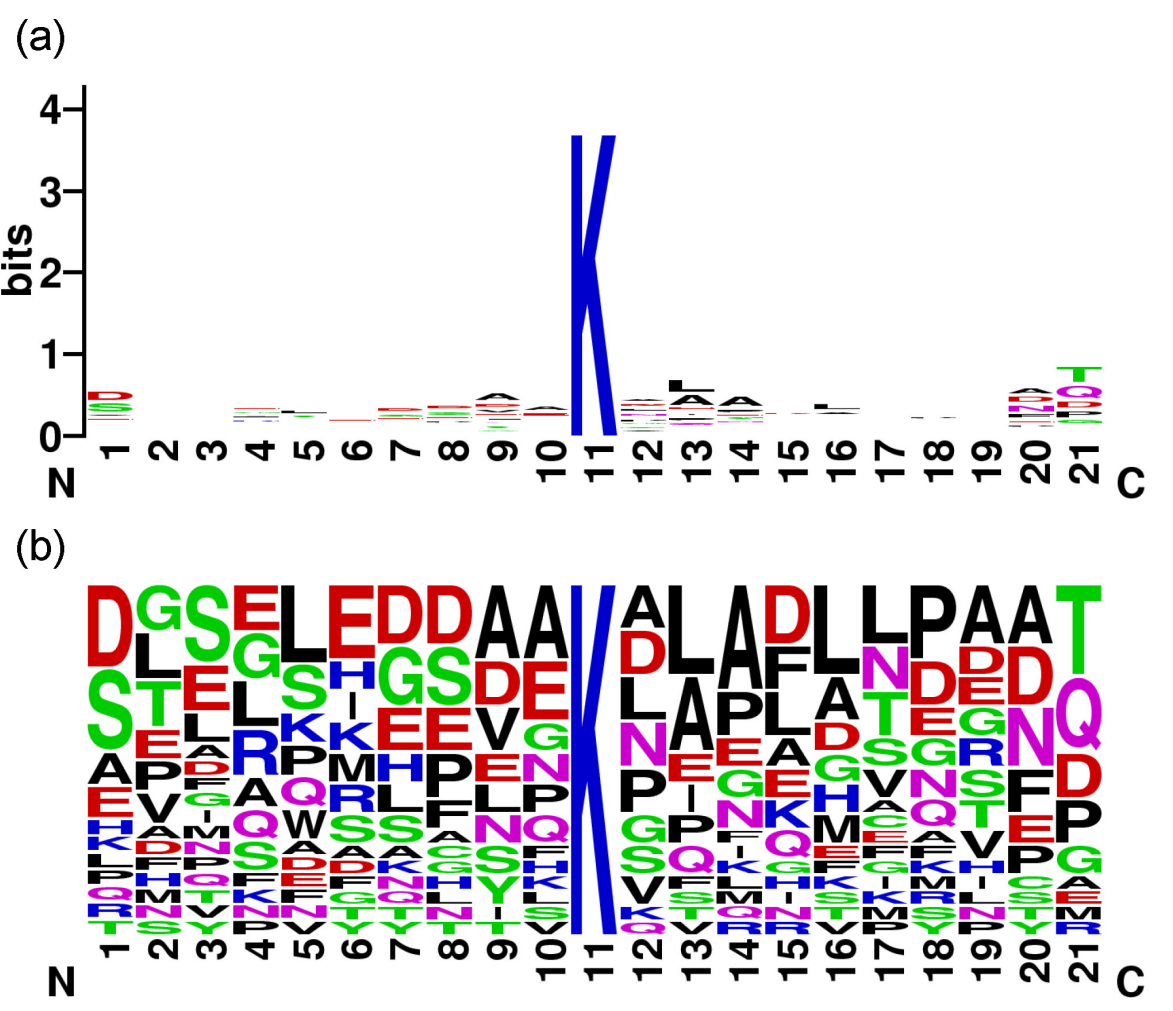
The sequence logo of the 23 peptides of promising ubiquitylation sites with *w *= 21. (a) Information content and (b) Frequency plot.

The prediction system UbiPred is applied to score 3424 putative non-ubiquitylation sites in an independent dataset that are not included in the training dataset of *w *= 21, as shown in Fig. 8. The screening result is shown in Fig. 9 using a histogram of prediction scores. There are 1218 putative non-ubiquitylation sites with scores larger than 0.5. There are 23 peptides with scores larger than 0.85, which are the most promising ubiquitylation sites, listed in Table 5. The detailed information can be found in the website of UbiPred [20]. The sequence logo of the 23 peptides shown in Fig. 10 represents low information content similar to the sequence logo of the 151 positive samples in training dataset.

**Table 5:** List of 23 promising ubiquitylation sites identified from an independent dataset of 3424 putative non-ubiquitylation sites.

Accession number	Position	Score	Accession number	Position	Score	Accession number	Position	Score
P19358	114	0.99	P39976	323	0.90	P38080	809	0.87
Q9Y6K9	35	0.96	P38261	147	0.89	P10592	54	0.87
P25694	6	0.96	P25360	846	0.89	P38080	792	0.87
P40087	325	0.95	P09936	195	0.88	P12866	129	0.86
Q08412	232	0.93	P10591	54	0.88	Q05911	460	0.86
P04629	609	0.91	Q06408	156	0.87	P40087	410	0.86
P16603	165	0.91	P37303	283	0.87	P38075	10	0.86
P31539	626	0.91	P32467	38	0.87			

For further validating the 23 peptides as ubiquitylation sites, the five prediction rules obtained from C5.0 (shown in Table 2) were applied to the 23 peptides. Results show that all the 23 promising peptides are classified as ubiquitylation sites. For example, the average value of property MEIH800102 for the 23 peptides is 1.001 which is larger than the threshold of 0.95. This value is close to that (1.007) of the 151 positive samples in training dataset. Note that the smallest and largest index values of MEIH800102 for 20 amino acids are 0.73 and 1.23, respectively. The prediction system UbiPred can predict ubiquitylation sites with prediction scores to identify the most promising ubiquitylation sites for experimental verification or future research.

## Conclusion

Ubiquitylation plays many important regulatory roles in the physiology of eukaryotic cell. Nowadays, many experimental studies are working on identifying ubiquitylated proteins and their ubiquitylation sites. To efficiently identify promising ubiquitylation sites by computational prediction methods is helpful to save experimental efforts. In this study, the combinations of three kinds of features (amino acid identity, evolutionary information, and all physicochemical properties) and three classifiers (support vector machine, *k*-nearest neighbor, and NaïveBayes) were evaluated for predicting ubiquitylation sites. The ubiquitylation dataset consists of 157 ubiquitylation sites and 3676 putative non-ubiquitylation sites extracted from 105 proteins in the UbiProt database. Results show that the best prediction method is the combination of using an SVM classifier and all physicochemical properties.

It is well recognized that irrelevant information will interfere with classifiers. This study proposes an algorithm IPMA to identify a small set of informative physicochemical properties to advance the prediction performance and further understand the underlying mechanism of ubiquitylation. The derived 31 informative physicochemical properties improve the prediction accuracy from 72.19% to 84.44%, and the properties were ranked in terms of their individual effectiveness of prediction. A decision tree method C5.0 was also applied to derive the rule-based knowledge and analyze the 31 informative physicochemical properties. Five concise rules provide a human-interpretable way to biologist for distinguishing ubiquitylation sites from non-ubiquitylation sites.

Finally, the system UbiPred for predicting ubiquitylation sites is designed by using the 31 informative physicochemical properties. The web server of UbiPred has been implemented and is available online [20]. The prediction scores of UbiPred can be utilized to identify promising ubiquitylation sites for experimental verification. In this study, 23 promising ubiquitylation sites whose prediction scores are larger than 0.85 were identified from an independent dataset of 3424 putative non-ubiquitylation sites and were also validated by the five concise rules obtained from the training dataset.

## Methods

### Establishment of datasets

To evaluate the two proposed methods IPMA and UbiPred, a positive dataset UBIDATA consisting of 157 ubiquitylation sites from 105 proteins was established by extracting annotated proteins from the UbiProt database [17]. By mapping the ubiquitylation sites to the corresponding 105 protein sequences retrieved from the UniProt Knowledgebase (Swiss-Prot and TrEMBL) [30], the 3676 lysine residues with no annotation of ubiquitylation sites were regarded as putative non-ubiquitylation sites. A sliding window method is applied to the central residue to be predicted for gleaning environment information. A positive sample is denoted as a sequence of size *w *with a central residue lysine which is an ubiquitylation site. If the central residue lysine is not an ubiquitylation site, the sequence is regarded as a negative sample. Only one of the samples with the same sequences and annotation of ubiquitylation sites was used. All the inconsistent samples which have the same sequences but not the same annotation were discarded. The 10 positive datasets were constructed using various values of *w *from UBIDATA, which have 149 samples of *w *= 11, 150 samples of *w *= 13 and 15, and 151 samples of *w *= 17, 19,..., 29. Due to the discard of duplicate and inconsistent samples, different values of *w *would result in different sample numbers of datasets.

For training an SVM classifier, both positive and negative samples are necessary. The dataset of post-translational modification including phosphorylation and ubiquitylation sites is unbalanced that the number of positive samples is much smaller than that of negative samples. The negative samples for training the SVM classifier were selected randomly from the 3676 putative non-ubiquitylation sites. In this study, the number of negative samples is the same with that of positive samples in the dataset. For example, there are 151 negative samples in the dataset of *w *= 21. The rest (e.g., 3424 samples with no annotation of ubiquitylation sites for *w *= 21) are formed as an independent dataset to be scored for identifying promising ubiquitylation sites (see Fig. 8). Notably, since the value of *C *for tuning the error penalty (see the next section) is determined subsequently according to the performance measurement of SVM, it is not obligatory to select a matched number of negative peptides for training the SVM classifier. The used datasets of various windows sizes can be publicly downloaded from the web server of UbiPred [20].

### Assessment of features and classifiers

Support vector machine (SVM) is a very popular and powerful method to deal with classification, prediction, and regression problems. To cope with the over-fitting problem arising from a small training dataset, SVM aims to find a linear separation hyperplane which maximizes the distance between two classes to create a classifier. Given training vectors **x**_*i *_∈ *R*^*n *^and their class values *y*_*i *_∈ {-1, 1}, *i *= 1,..., *N*, SVM solves the problem of minimizing $\frac{1}{2}w^{\text{T}}w+C\sum_{i=1}^{N}\xi_{i}$, subject to *y*_*i *_(**w**^T ^**x**_*i *_+ *b*) ≥ 1 - *ξ*_*i *_and *ξ*_*i *_≥ 0, where **w **is a normal vector perpendicular to the hyperplane and *ξ*_*i *_are slake variables for allowing misclassifications. The cost parameter *C *(> 0) controls the trade-off between the margin and the training error. Larger value of *C *will lead to a higher error penalty. The kernel function of SVM transforms samples to a high-dimensional space to make linear separation easier. The commonly-used radial basis kernel function is applied to non-linearly transform the feature space, defined as *K*(*x*_*i*_, *x*_*j*_) = exp(-*γ*||*x*_*i *_- *x*_*j*_||), where *γ *> 0 is the kernel parameter, deciding how the samples are transformed to a high-dimensional space. These two parameters (*C *and γ) must be tuned to obtain satisfactory prediction results. In this study, the used SVM package is LIBSVM of version 2.84 [31].

Two extensively used classifiers, the *k*-nearest neighbor classifier (IBk) and the NaïveBayes classifier that are included in the machine learning tool WEKA [32], are also utilized to evaluate the promising prediction features. To obtain the best performance, five versions of the IBk classifier with *k *= 1, 3,..., 9 are evaluated for identifying the best value of *k*. For the NaïveBayes classifier, in addition to normal distribution, a distribution obtained from a kernel density estimator is used to model numeric attributes [32].

Informative features will lead to better performances of classifiers. Numerous features can be extracted from peptide sequences [11-16]. This study assesses three kinds of features including amino acid identity, evolutionary information, and physicochemical property. The feature representations used for the above-mentioned classifiers are described below.

The conventional feature representation of amino acid identity uses 20 binary bits to represent an amino acid [11,13]. For example, the amino acid A is represented by '00000000000000000001' and R is represented by '00000000000000000010'. To deal with the problem of windows spanning out of N-terminal or C-terminal, one additional bit is appended to indicate this situation. A vector of size (20+1)*w *bits is used for representing a sample.

Evolutionary information has been successfully used in many studies [14,15]. To prepare evolutionary information for each protein sequence, the corresponding position-specific scoring matrix (PSSM) is obtained by applying PSI-BLAST [33] against non-redundant SWISS-PROT database using 3 iteration and default values of parameters. The matrix has 20*L elements, where *L *is the length of a peptide. For each residue, there are 20 values indicating the probabilities of occurrences for 20 amino acids. By using the window size *w*, there are 20*w elements to represent a peptide [14,15]. One additional bit is utilized to deal with the terminal spanning windows as used for amino acid identity [14,15]. Therefore, a vector of size (20+1)*w *is used for representing a sample.

Physicochemical property is the most intuitive feature for biochemical reactions and is extensively applied in bioinformatics studies. The amino acid indices (AAindex) database collects many published indices representing physicochemical properties of amino acids. For each physicochemical property, there is a set of 20 numerical values for amino acids. Currently, 544 physicochemical properties can be retrieved from the AAindex database of version 9.0 [34]. After removing physicochemical properties having the value 'NA' in the amino acid indices, 531 physicochemical properties are obtained for the following studies. In contrast to the residue-based encoding methods of amino acid identity and evolutionary information, a vector of 531 mean values is used to represent a sample for various window sizes [12,16]. The method of encoding the input vector from peptide sequences consists of two steps. First, a vector of 531 index values is determined for each amino acid of the peptide. For a peptide of size *w*, there are *w *531-dimensional vectors. Notably, the number of amino acids for the peptide with a terminal spanning window would be smaller than *w*. The second step is to construct a vector of 531 mean values obtained by averaging these 531-dimensional vectors [12,16]. If *m *out of 531 informative physicochemical properties are selected by IPMA and are used in SVM, a vector of *m *mean values is used to represent a sample.

To find the best features for the SVM-based method, the control parameters *C *and γ of SVM and associated window size *w *∈ {11, 13,..., 29} should be tuned for each kind of features. The grid search method is applied to tune the parameters *C *and γ ∈ {2^-7^, 2^-6^,..., 2^8^} that a total number 256 (= 16*16) of grids are evaluated. The prediction accuracy of 10-CV is used to determine the best features and classifier.

### Informative physicochemical property mining algorithm

An informative physicochemical property mining algorithm (IPMA) is proposed to select a small set of *m *informative physicochemical properties form a large set of *n *= 531 physicochemical properties and determine the values of *C *and γ of the used SVM simultaneously. The IPMA is based on an inheritable bi-objective genetic algorithm (GA) [18] which is an efficient method for solving the bi-objective 0/1 combinatorial optimization problem C(*n*, *m*). In using the IPMA, minimizing the number *m *of properties (features) and maximizing the prediction accuracy are the two objectives to be achieved. High performance of the inheritable bi-objective GA arises mainly from an intelligent evolutionary algorithm [35] which can efficiently solve large-scale parameter optimization problems by using a divide-and-conquer strategy and orthogonal array crossover with a systematic reasoning method instead of traditional generate-and-go in the crossover operation.

The encoded GA-chromosome X consists of *n *= 531 bits for selecting physicochemical properties (1 for inclusion and 0 for exclusion) and two 4-bit GA-genes for tuning parameters *C *and γ of SVM. The two 4-bit GA-genes map the 16 values of *C *and γ into {2^-7^, 2^-6^,..., 2^8^}. IPMA can simultaneously obtain a set of solutions X_r _to C(*n*, *r*) where *r *= *r*_start_, *r*_start _+1,..., *r*_end _in a single run. The best among all X_r _according to the fitness function *f*(X) is the desirable solution X_*m *_where *f*(X) is the overall accuracy of 10-CV. By decoding X_*m*_, *m *informative physicochemical properties and the SVM classifier can be obtained at the same time.

The algorithm IPMA with the given values of *r*_start _and *r*_end _is described below. In this study, the used parameters of IPMA are *N*_pop _= 50, *P*_c _= 0.8, *P*_m _= 0.05, *r*_start _= 5, and *r*_end _= 45 according to experience.

Step 1) (Initiation) Randomly generate an initial population of *N*_pop _individuals. All the *n *binary genes have *r *1's and *n-r *0's where *r *= *r*_start_.

Step 2) (Evaluation) Evaluate the fitness values of *f*(X) for all individuals.

Step 3) (Selection) Use the traditional tournament selection that selects the winner from two randomly selected individuals to form a mating pool.

Step 4) (Crossover) Select *P*_c_·*N*_pop _parents from the mating pool to perform orthogonal array crossover [35] on the selected pairs of parents where *P*_c _is the crossover probability.

Step 5) (Mutation) Apply a bit-inverse mutation operator with a mutation probability *P*_m _to the population by keeping the *n *binary parameters in an individual having *r *1's. To prevent the best fitness value from deteriorating, mutation is not applied to the best individual in the population (*I*_best_).

Step 6) (Termination test) If *I*_best _is not improved in 10 generations continuously, output *I*_best _as X_r_. Otherwise, go to Step 2).

Step 7) (Inheritance) If *r *<*r*_end_, randomly change one bit in the binary genes for each individual from 0 to 1; increase the number *r *by one, and go to Step 2). Otherwise, stop the algorithm.

### Rule-based knowledge acquirement

Decision tree methods are useful algorithms to acquire interpretable rule-based knowledge as well as classification of ubiquitylation sites. In this study, the decision tree method C5.0, an improved version of C4.5 [19], with rather high prediction accuracy, is applied to construct decision tree classifiers and derive interpretable rules. For C5.0, the information gain is utilized to rank features for constructing a decision tree by iteratively appending nodes with high ranks. The decision tree method can serve as a tool of feature selection by using the ranks of features. However, the set of selected features is constructed by considering individual effects of classification only but no correlation among relevant features.

To avoid over-fitting problems, a pruning process is applied to reduce the tree size by replacing a subtree with a leaf node. The used threshold value of confidence for pruning trees is set to 25%. The final decision tree can directly generate if-then rules where one leaf node corresponds to one rule. The samples in the leaf node are the covered samples of this rule. The majority rule determines the class label. The samples with a relative small size in the leaf node are regarded as misclassified samples. To derive more simple rule-based knowledge, the option '-r' of C5.0 is applied to generate rules of small length for intuitive interpretation.

## Authors' contributions

CWT designed the system, implemented programs, developed the web server, carried out the analysis, and participated in manuscript preparation. SYH supervised the whole project and participated in manuscript preparation. All authors have read and approved the final manuscript.
